# Phospholipid-Based Delivery System Optimizes the Solubility and Systemic Exposure of Palmitoylethanolamide and Supports Clinical Benefits in Chronic Neuropathic Low Back Pain

**DOI:** 10.3390/biomedicines14020380

**Published:** 2026-02-06

**Authors:** Amjad Khan, Fazle Rabbani, Ayesha Kanwal, Areaba Shafiq, Ikram Ujjan, Anna Vellaccio, Massimo Ronchi, Giovanna Petrangolini, Eric De Combarieu, Silvia Turroni, Gabriele Conti

**Affiliations:** 1 Nuffield Division of Clinical Laboratory Sciences (NDCLS), Radcliffe Department of Medicine, University of Oxford, John Radcliffe Hospital, Headley Way, Oxford OX3 9DU, UK; 2 Department of Biochemistry, Liaquat University of Medical & Health Sciences (LUMHS), Jamshoro 76090, Pakistan; 3 Department of Psychiatry, Lady Reading Hospital (LRH)-MTI, Peshawar 25000, Pakistan; ayesha.kanwal@lrh.edu.pk (A.K.); areaba.shafiq@lrh.edu.pk (A.S.); 4 Department of Pathology, Liaquat University of Medical & Health Sciences, Jamshoro 76090, Pakistan; 5 Medical, Formulation Development and R&D Departments, Indena, S.p.A, Viale Ortles, 12, 20139 Milan, Italy; 6 Unit of Microbiome Science and Biotechnology, Department of Pharmacy and Biotechnology, University of Bologna, 40126 Bologna, Italy; 7 Human Microbiomics Unit, Department of Medical and Surgical Sciences, University of Bologna, 40126 Bologna, Italy; gabriele.conti12@unibo.it

**Keywords:** palmitoylethanolamide, neuropathic pain, low back pain, nutraceutical, Phytosome™, clinical trial, Cronilief™

## Abstract

**Background:** Chronic neuropathic low back pain (LBP) is a prevalent health condition and difficult to treat. Conventional therapies often provide limited relief and raise safety concerns. Supplemental palmitoylethanolamide (PEA), an endogenous fatty acid amide with analgesic and anti-inflammatory properties, has shown benefits in neuropathic pain, but its application as a supportive strategy has been limited by poor oral bioavailability. **Objectives**: This study aimed to investigate a phospholipid-based palmitoylethanolamide formulation (PEA-PL, Cronilief™), developed using Phytosome™ delivery technology, with respect to solubility optimization, systemic exposure, and associated clinical effects in individuals with chronic neuropathic LBP. **Methods:** PEA-PL solubility was assessed in fasted-state simulated intestinal fluid and compared with unformulated PEA. Plasma PEA concentrations were evaluated in healthy volunteers after 2 weeks of supplementation with unformulated PEA (300 mg/day) or PEA-PL (300 or 600 mg/day). Clinical efficacy was assessed in a double-blind, placebo-controlled randomized, trial in which 120 adults with neuropathic LBP received PEA-PL 600 → 300 mg (*n* = 40), PEA-PL 450 mg (*n* = 40), or placebo (*n* = 40), daily for 8 weeks in addition to Standard of Care. Primary outcomes were effects on neuropathic pain (Douleur Neuropathique 4, DN4) and its intensity (Numeric Pain Rating Scale, NPRS). Secondary outcomes included effect on functional disability (Oswestry Disability Index, ODI), sleep quality (Pittsburgh Sleep Quality Index, PSQI), quality of life (QoL) (SF-12), and concomitant analgesic use. Safety was monitored throughout the 8-week supplementation period. **Results:** PEA-PL increased PEA solubility approximately eight-fold and resulted in higher plasma PEA concentrations than unformulated PEA. Both PEA-PL regimens significantly improved pain, functional disability, sleep, and QoL outcomes versus placebo (all *p* < 0.0001), with greater effects for the 600 → 300 mg regimen. Analgesic discontinuation occurred more frequently in PEA-PL groups (65–70%). Supplementation was well tolerated. **Conclusions:** A phospholipid-based (Phytosome™) PEA formulation (Cronilief™) was developed and associated with optimized systemic exposure and clinically meaningful reductions in pain severity and functional disability in individuals with chronic neuropathic LBP.

## 1. Introduction

Low back pain (LBP) is a highly prevalent health condition experienced by most individuals at some point during their lifetime and represents one of the leading causes of chronic pain worldwide. In 2020, approximately 619 million people were affected by LBP globally, and this number is projected to increase to 843 million by 2050 [1]. Clinically, LBP encompasses a spectrum of pain phenotypes, including a substantial neuropathic component in a relevant proportion of patients. LBP is defined as pain, either acute or chronic (when persisting for more than 12 weeks), localized between the lower margin of the ribs and the gluteal folds. In some cases, pain may radiate to the lower limbs and be accompanied by muscle weakness or sensory disturbances, such as numbness [2]. Chronic LBP is frequently associated with comorbidities including depression, anxiety, and sleep disturbances, thereby substantially increasing both individual suffering and societal burden [3,4]. The lifetime prevalence of LBP exceeds 70% in industrialized countries, and longitudinal evidence from prospective cohort studies indicates that up to two-thirds of affected individuals progress to chronic LBP [3,4].

Chronic LBP is a heterogeneous condition driven by overlapping nociceptive and neuropathic mechanisms [5]. Although clinical guidelines estimate that neuropathic pain affects approximately 5% of individuals with LBP, accumulating evidence suggests that neuropathic components may be present in 16–55% of patients with chronic LBP [3]. Despite this, the neuropathic component of chronic LBP is frequently underestimated in clinical practice. Early identification and targeted treatment are therefore crucial for effective disease management [5]. The likelihood of neuropathic involvement can be assessed using validated clinical screening tools, such as the Douleur Neuropathique en 4 (DN4) questions, in subjects with LBP [6]. Importantly, the presence of a neuropathic component may exacerbate the overall pain syndrome [2], leading to reduced mobility and impairments in work, social functioning, sleep quality, low mood, and depression [3].

Current pharmacological treatment of chronic LBP includes paracetamol (acetaminophen), nonsteroidal anti-inflammatory drugs (NSAIDs), antidepressants, anticonvulsants, opioids, and topical therapies [3]. In subjects with a neuropathic component, these treatments are often limited by modest efficacy, incomplete or unsustainable pain relief, and adverse effects that restrict long-term use [7,8,9,10]. Consequently, contemporary clinical guidelines emphasize an evidence-based, multimodal approach that minimizes prolonged reliance on pharmacological treatments while balancing therapeutic benefits with patient safety and quality of life (QoL). In this context, there is a need for adjunctive strategies that can be safely integrated into long-term management to address pain and its associated functional consequences.

Owing to generally favorable safety and tolerability profiles, and the ability to exert analgesic and anti-inflammatory effects across multiple biological pathways [11,12], dietary supplementation has attracted increasing clinical interest as a supportive approach for chronic pain management.

Palmitoylethanolamide (PEA) is an endogenous lipid mediator belonging to the N-acyl-ethanolamine (NAE) fatty acid amide family, with cannabimimetic properties, that has been increasingly investigated for its role in the management of chronic and neuropathic pain [13]. First isolated from soybean lecithin and egg yolk in 1957, PEA was initially recognized for its anti-inflammatory and anti-allergic effects [14]. PEA modulates pain pathways through multiple receptor-mediated and indirect mechanisms [13], primarily via activation of the peroxisome proliferator-activated receptor-α (PPAR-α) [15]. Activation of PPAR-α leads to downstream suppression of inflammatory signaling pathways, including inhibition of nuclear factor kappa-light-chain-enhancer of activated B cells (NF-κB) nuclear translocation and reduced production of pro-inflammatory mediators such as interleukins, tumor necrosis factor-α (TNF-α), and prostaglandins [16]. In addition, PEA downregulates mast cell degranulation and microglial activation, which are key contributors to peripheral and central sensitization [17]. Further mechanisms include modulation of transient receptor potential vanilloid 1 (TRPV1) channel and G protein-coupled receptors 55 (GPR55), as well as indirect enhancement of endocannabinoid signaling through inhibition of fatty acid amide hydrolase—commonly referred to as the “entourage effect” [15,18]. The convergence of these mechanisms positions PEA as a multi-target compound in the context of chronic and neuropathic pain. By attenuating neuroinflammation and reducing nociceptor sensitization, PEA may exert effects that extend beyond symptomatic pain relief [18].

Evidence from several randomized controlled trials and observational studies has reported beneficial effects of PEA supplementation across a range of conditions, including peripheral neuropathic pain, musculoskeletal disorders, and palliative care settings [19,20,21,22]. Consistently, a recent meta-analysis has shown that PEA supplementation in chronic pain is associated with significant improvements in pain intensity, physical function, and QoL [20].

Despite these promising findings, the lipophilic nature of PEA and its poor water solubility limit its oral bioavailability and, consequently, the development of nutraceutical applications. The principal rate-limiting factors affecting PEA absorption include its slow dissolution kinetics and the predominantly hydrophilic nature of the intestinal lumen [23], which limits solubilization of lipophilic compounds. Following absorption, PEA is rapidly metabolized and excreted [18,21]. To address these limitations, several formulation delivery techniques—such as emulsification, micronization, and incorporation into specialized dispersion systems—have been utilized to improve the oral performance of PEA in dietary supplements [18,23,24,25].

In the present study, a phospholipid-delivery system–based (Phytosome™) formulation of PEA was evaluated for its solubility, systemic exposure, and clinical performance in individuals with chronic neuropathic LBP. This phospholipid-based delivery system has been previously utilized for various natural bioactive compounds, including quercetin, berberine, curcumin, boswellic acids, lemon balm extracts, and coenzyme Q10, in which its amphipathic nature facilitates wetting and limits self-aggregation, thereby enhancing solubility, optimizing intestinal absorption, and supporting formulation stability [26,27,28,29,30,31,32].

## 2. Materials and Methods

### 2.1. Preparation of Phospholipids-Delivery System-Based PEA Formulation (PEA-PL)

Palmitoylethanolamide (PEA) was obtained from commercially available palmitic acid certified by the Roundtable on Sustainable Palm Oil (RSPO; Kuala Lumpur, Malaysia). PEA-PL (Cronilief™, Indena S.p.A., Milan, Italy) represents an oral formulation of PEA produced using a phospholipid-based dispersion technology (Phytosome™), which is employed to enhance the solubility and gastrointestinal passage of lipophilic bioactive compounds. Cronilief™ was prepared using a solvent evaporation method. Briefly, PEA, sunflower lecithin, and microcrystalline cellulose were solubilized and dispersed in an organic solvent. The solvent was subsequently removed under reduced pressure, and silicon dioxide was added to the resulting dry powder to improve flowability. A final granulation step was performed to obtain the desired particle size distribution. The resulting PEA-PL was standardized to contain 35–40% (*w*/*w*) PEA, as determined by high-performance liquid chromatography (HPLC) against PEA external standard on H-Class system with Photodiode Array (PDA) detector from Waters equipped with a Zorbax Eclipse XDB-C8 (5 µm) column (250 × 4.6 mm I.D.) from Agilent Technologies. A step gradient of water (A) and acetonitrile (B) was used at 1.2 mL/min: 0–5 min, 42–35% A; 5–7 min, 35–8% A; 7–17 min, 8–0% A; 17–22 min, isocratic 100% B. Column temperature was set to 40 °C and UV detection at 202 nm. About 25 mg, accurately weighed, of the sample is solubilized in 50 mL of methanol and filtered before HPLC injection (10 µL).

### 2.2. Formulation Solubility Study

Samples of the PEA-PL and unformulated PEA (Frau Pharma, Agrate Brianza, MB, Italy), each corresponding to 10 mg of PEA, were incubated under magnetic stirring in 20 mL of fasted-state simulated intestinal fluid (FaSSIF; Biorelevant, QMB Innovation Centre, 42 New Road, London, UK) at pH 6.5 for 2 h at room temperature. Following incubation, samples were filtered through a 0.45 µm hydrophilic polyvinylidene fluoride (PVDF) membrane filter to obtain clear solutions, which were subsequently analyzed by HPLC. The HPLC system consisted of a quaternary pump, a thermostatic column compartment, and a UV/Vis detector. Chromatographic separation was achieved using an Agilent Zorbax Eclipse XDB-C8 column (250 × 4.6 mm, 5 µm particle size) from Agilent Technologies, Santa Clara, USA. The mobile phase comprised water (solvent A) and acetonitrile (solvent B), delivered at a flow rate of 1.2 mL/min using a linear gradient as follows: 42% A at 0 min; 35% A at 5 min; 8% A at 7 min; 0% A at 17 min; 0% A at 22 min; and 42% A at 23 min. The column temperature is set to 40 °C and the autosampler temperature is maintained at 25 °C; detection was performed at 202 nm. Data were finally expressed as mean ± standard deviation (SD).

### 2.3. PEA Plasma Concentration Study in Healthy Adults

A randomized, six-sequence, three-period crossover exploratory clinical study (3 × 3 × 3 design with balanced carryover effects) was conducted to evaluate plasma concentrations of PEA in healthy adult volunteers following supplementation with PEA-PL compared with unformulated PEA. The study was performed at the Bioaraba Clinical Trials Unit (CTU) of Araba University Hospital and Txagorritxu Hospital (Vitoria-Gasteiz, Spain). The study protocol was approved by the Research Ethics Committee of Araba University Hospital (Study code: 2022/028–IDN_PEAF01–22; approval date: 24 March 2023) and was conducted in accordance with the Declaration of Helsinki, the principles of Good Clinical Practice (GCP), Directive 2005/28/EC, and the Standard Operating Procedures (SOPs) of the CTU, which are based on the ICH-GCP guidelines. Written informed consent was obtained from all participants prior to study initiation.

Twelve participants who met the predefined inclusion and exclusion criteria were enrolled. Inclusion criteria were male or female subjects aged 18–50 years (inclusive); body mass index (BMI) between 18.5 and 27 kg/m^2^; absence of clinically significant organic or psychiatric disease; negative serology for hepatitis B virus (HBV), hepatitis C virus (HCV), and human immunodeficiency virus (HIV); and vital signs and electrocardiogram (ECG) findings within normal limits. Exclusion criteria included: current or history of alcohol or drug abuse; known allergy or hypersensitivity to PEA or any formulation components; regular use of prescription medications, over-the-counter drugs, or herbal products within 15 days prior to study initiation; intake of any PEA-containing drug or dietary supplement within 30 days before study entry; pregnancy or breastfeeding. In addition, participants received a list of PEA-containing foods with specified maximum permitted intake levels and were instructed to comply with these dietary restrictions from at least 72 h prior to Day 1 until completion of the study.

Unformulated PEA (300 mg) and PEA-PL (300 mg, corresponding to 120 mg of PEA) were manufactured as indistinguishable film-coated tablets. Tablets contained the following food-grade excipients: dicalcium phosphate dihydrate, microcrystalline cellulose, polyvinylpolypyrrolidone, silicon dioxide, talc, magnesium stearate, and a titanium dioxide–free film coating. Prior to release, tablets were analyzed for PEA content by HPLC, as well as for appearance, average mass, mass uniformity, disintegration time, and microbiological quality. Given the crossover design, each participant served as his or her own control, and no independent parallel control group was included. Study products were administered orally once daily at breakfast according to the assigned treatment sequence: (i) unformulated PEA, 300 mg (one film-coated tablet); (ii) PEA-PL, 300 mg (one film-coated tablet, equivalent to 120 mg of PEA); (iii) PEA-PL, 600 mg (two film-coated tablets, equivalent to 240 mg of PEA). Each treatment was administered for three periods of 2 weeks each, with a washout period of at least 3 weeks between periods to minimize potential carryover effects. The treatment sequence was assigned using a predefined randomization list.

Blood samples (8 mL) were collected from each participant at three time points during each study period: baseline (pre-dose, T0), 2 h after the first dose (T1), and 2 h after the last dose following 2 weeks of supplementation (T2). Plasma was separated into two aliquots and stored at −80 °C until analysis. Due to the presence of endogenous PEA, calibration curves and quality control samples were prepared using synthetic plasma (3% bovine serum albumin in phosphate-buffered saline; Sigma-Aldrich). PEA quantification was performed on 200 µL plasma samples (K2-EDTA anticoagulant) using a validated LC-MS/MS method following protein precipitation with acetonitrile (Scharlau). PEA-d4 (Cayman Chemical, Ann Arbor, MI, USA) was used as the internal standard.

Analyses were performed by Kymos Pharma Services S.L. (Barcelona, Spain) in accordance with U.S. Food and Drug Administration (FDA) and European Medicines Agency (EMA) bioanalytical method validation guidelines. The calibration range was 100–8000 pg/mL, with a lower limit of quantification (LLOQ) of 100 pg/mL. The method was selective, sensitive, accurate, and precise within the validated range. Total plasma PEA concentrations were used for the analyses on absorption levels.

Clinical safety was assessed by monitoring adverse events and vital signs, whereas biological safety was evaluated through hematological and biochemical parameters.

Intra-group differences were evaluated using either the Wilcoxon signed-rank test or two-tailed paired Student’s *t*-test, as appropriate. Inter-group comparisons were performed using two-way repeated-measures analysis of variance (ANOVA), followed by Sidak’s post hoc test when applicable. Statistical significance was set at *p* < 0.05.

### 2.4. Clinical Efficacy Study in Chronic Neuropathic Low Back Pain

This was a single-center, prospective, double-blind, placebo-controlled randomized clinical trial conducted at the outpatient clinic of Lady Reading Hospital (LRH), Peshawar, Pakistan. Participants were enrolled between January and March 2025. The trial evaluated the potential analgesic efficacy and safety of different dose regimens of PEA-PL compared with placebo, administered as an add-on to Standard of Care (SoC) in adults with a moderate degree of chronic LBP with a neuropathic component. The study was approved by the Research Ethics Committee of Liaquat University of Medical & Health Sciences (Ref. No. LUMHS/REC/531/25.11.2024) and the LRH Institutional Review Board (Ref. No. 528/LRH/MTI/12.12.2024) Pakistan, conducted in accordance with the Declaration of Helsinki and GCP guidelines, and registered at ClinicalTrials.gov (Identifier: NCT06694337). Written informed consent was obtained from all participants prior to enrolment.

Eligible participants were adults aged 18–80 years with a moderate degree of chronic neuropathic LBP persisting for at least 3 months. The neuropathic component was established by clinical evaluation supported by the DN4 questionnaire, a validated diagnostic tool consisting of 10 items grouped into four questions assessing both sensory descriptors and clinical examination findings [6,33,34]. Each affirmative response is scored as 1 point (maximum score: 10); a DN4 score ≥ 4 indicates likely neuropathic pain.

Participants were also required to have moderate pain intensity, defined as a score of 4–6 on the Numeric Pain Rating Scale (NPRS or NRS), an 11-point self-reported scale ranging from 0 (“no pain”) to 10 (“worst imaginable pain”) [34,35,36]. Exclusion criteria included significant systemic or organic disease; current or history of alcohol or drug abuse; history of malignancy within the previous 5 years; pregnancy or breastfeeding; and severe psychiatric disorders (e.g., major depression or schizophrenia) that could affect pain perception or study adherence. Individuals with opioid dependence, those using cannabinoid-based therapies, or those currently taking dietary supplements with potential analgesic or anti-inflammatory effects (including omega-3 fatty acids, turmeric/curcumin, glucosamine, chondroitin, Boswellia, and methylsulfonylmethane) were excluded.

Following baseline assessment, participants were randomized in a 1:1:1 ratio to receive one of the following interventions for 8 weeks, in addition to SoC: PEA-PL 600 → 300 mg, PEA-PL 450 mg, or placebo, to be taken after a meal. Randomization was carried out using a computer-generated sequence prepared by an independent statistician, and treatment allocation was concealed using sequentially coded, identical study products. Supplementation regimens were as follows:

- PEA-PL 600 → 300 mg: Week 1, two tablets/day (600 mg/day); Weeks 2–8, one tablet/day (300 mg/day).
- PEA-PL 450 mg: Week 1, one active tablet (450 mg/day) plus one placebo tablet; Weeks 2–8, one active tablet/day (450 mg/day).
- Placebo: Week 1, two tablets/day; Weeks 2–8, one tablet/day.

SoC was continued as needed throughout the trial and included commonly prescribed analgesics such as paracetamol and NSAIDs, e.g., ibuprofen, naproxen, diclofenac, administered orally or topically. Supportive therapies, including massage and physiotherapy, were permitted. All concomitant treatments were documented. Participants maintained daily diaries documenting supplement intake, SoC medication use, and adverse events. Compliance was assessed through weekly telephone follow-up and pill counts at study visits.

Baseline demographic and clinical data—including age, sex, ethnicity, body mass index (BMI), vital signs, medical history, and concomitant medications—were recorded in the Case Report Form (CRF).

Clinical assessments were conducted at baseline and at Weeks 1, 4, and 8. The following outcome measures were collected:

- DN4: baseline and Week 8
- NPRS: baseline, Week 1, Week 4, and Week 8
- Oswestry Disability Index (ODI): baseline, Week 1, Week 4, and Week 8
- Short Form Health Survey–12 (SF-12): baseline and Week 8
- Pittsburgh Sleep Quality Index (PSQI): baseline, Week 4, and Week 8

The study’s primary outcome was the change in the neuropathic pain as experienced by the subject (DN4) and its severity (NPRS) from baseline to Week 8. Secondary outcomes included functional disability, health-related QoL, and sleep quality. Functional disability was measured with the ODI, a self-administered, 10-item questionnaire widely regarded as the gold standard for assessing disability in LBP [37]. Each item is scored from 0 to 5, with total scores converted to percentages to classify disability as minimal (0–20%), moderate (21–40%), severe (41–60%), very severe (61–80%), or complete (81–100%). Health-related QoL was assessed using the Short Form Health Survey-12 (SF-12), a self-administered 12-item questionnaire that captures eight domains of health: physical functioning, role limitations due to physical health, bodily pain, general health, vitality, social functioning, role limitations due to emotional health, and mental health. It provides two composite scores: the Physical Component Summary (PCS) and the Mental Component Summary (MCS) [38]. Sleep quality was evaluated with the PSQI, an 18-item, self-administered questionnaire assessing seven domains of sleep over the previous 4 weeks, with total scores ranging from 0 to 21; scores ≥ 5 indicate poor sleep quality [39].

Safety and tolerability were evaluated through monitoring of adverse events and participant-reported symptoms throughout the 8-week supplementation period.

A sample size of 120 participants (40 per group) was considered appropriate for this exploratory trial, based on previously reported randomized studies of PEA in chronic LBP with a neuropathic component [19,20]. 

Demographic and baseline characteristics were summarized as mean ± standard error (SEM) for continuous variables and as counts and percentages for categorical variables. Between-group comparisons at baseline were assessed using the Kruskal–Wallis test for continuous variables and χ^2^ or Fisher’s exact test for categorical variables. Longitudinal outcomes (DN4, NPRS, ODI, PSQI, and QoL ) and vital signs for safety assessment were analyzed using linear mixed-effects models (*lme4* and *lmerTest* R packages, R v4.5.0) [40,41] with study group, visit, and their interaction as fixed effects, and a random intercept for each participant. Gender, age, and BMI were included as covariates. Omnibus significance was assessed by Type III ANOVA with Satterthwaite’s approximation, and post hoc pairwise comparisons of estimated marginal means were conducted using *emmeans* [42] with *p*-values adjusted for multiple testing by the Benjamini–Hochberg false discovery rate (FDR). From the QoL survey, PCS and MCSscores were computed using the *lbscorer* package [38]. Results are reported as T-scores (mean = 50, SD = 10). Graphical outputs were generated in R (v4.5.0) using *ggplot2* and *microbAIDeR* [43,44] showing group means with SEM and clinically relevant thresholds (e.g., DN4 ≥ 4, PSQI ≥ 5, QoL PCS/MCS 50 ± 10). For QoL, principal component analysis (PCA) was performed, and group- and time-dependent shifts were tested using PERMANOVA (*pairwise.adonis2*, 9999 permutations). Multinomial logistic regression (*multinom* function of *nnet* R package) adjusted for Gender, Age category, and BMI category; global arm effect by LRT; pairwise *emmeans* contrasts reported as ORs (95% CI) with FDR correction [45]. Statistical significance was set at FDR-adjusted *p* ≤ 0.05, with trends reported at *p* ≤ 0.1. Significance levels were indicated in figures using the conventional star system (*p* ≤ 0.1, °; *p* ≤ 0.05, *; *p* ≤ 0.01; *p* ≤ 0.001, ***; *p* ≤ 0.0001,****).

## 3. Results

### 3.1. Formulation Solubility 

As shown in Table 1, phospholipid-based palmitoylethanolamide (PEA-PL) exhibited an approximately eight-fold higher solubility in FaSSIF, (pH 6.5, compared with unformulated PEA (0.133 vs. 0.016 mg/mL).

### 3.2. PEA Plasma Concentration in Healthy Adults

A total of 19 subjects were screened for eligibility. Of the 16 subjects who initiated the study, 11 completed all treatment periods. Five participants did not complete the study: three withdrew for personal reasons, and two discontinued due to mild to moderate gastrointestinal discomfort reported during supplementation.

No clinically relevant differences were observed among treatment periods with respect to vital signs, physical examination findings, or electrocardiographic parameters, indicating favorable tolerability of both unformulated PEA and PEA-PL.

As shown in Table 2, administration of unformulated PEA (300 mg/day) resulted in a significant increase in plasma PEA concentrations 2 h after the first dose compared with baseline (T1 vs. T0), rising from 1797.8 pg/mL to 2459.9 pg/mL (+37%, *p* < 0.05). After 2 weeks of supplementation, plasma PEA concentrations further increased to 3024.0 pg/mL (+68% vs. T1, *p* < 0.05). Administration of PEA-PL at 300 mg/day (equivalent to 120 mg of PEA) also produced a significant increase in plasma PEA concentrations 2 h after the first dose, from 1727.1 pg/mL at baseline to 2168.4 pg/mL (+26%, *p* < 0.05). After 2 weeks of supplementation, plasma PEA concentrations reached 3366.5 pg/mL, corresponding to an increase of approximately 95% relative to baseline (*p* < 0.01). When two tablets of PEA-PL were administered daily (600 mg/day, equivalent to 240 mg of PEA), a greater increase in plasma PEA concentrations was observed 2 h after the first dose, rising from 1963.2 pg/mL at baseline to 3244.5 pg/mL (+65%, *p* < 0.01). After 2 weeks, plasma PEA concentrations remained elevated at 3264.6 pg/mL (+66% vs. T1, *p* < 0.05).

**Figure 1:**
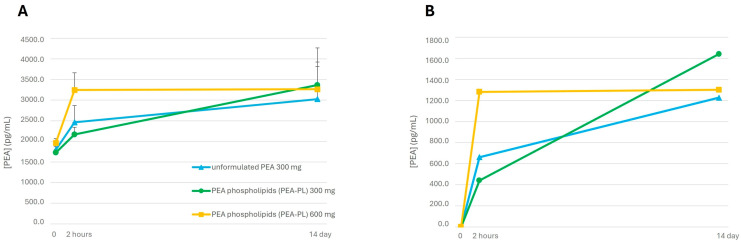
Healthy adults’ plasma palmitoylethanolamide (PEA) concentrations determined by LC–MS/MS following supplementation with unformulated PEA 300 mg (blue), PEA-PL 300 mg (green), and PEA-PL 600 mg (two 300 mg tablets; yellow) during a 2-week supplementation period. In the PEA-PL, the declared PEA content corresponds to 40% (*w*/*w*) of the total formulation weight. PEA concentrations are reported as mean ± SEM at baseline, 2 h after the first dose, and 2 h after the last dose of the 14-day regimen. (**A**) Absolute plasma PEA concentrations. (**B**) Plasma PEA concentrations normalized to baseline endogenous PEA levels.

As illustrated in Figure 1A, all PEA regimens resulted in progressive increases in plasma PEA concentrations over the 2-week period. When plasma concentrations were normalized to baseline endogenous PEA levels (Figure 1B), supplementation with PEA-PL yielded consistently higher relative increases compared with unformulated PEA. After additional normalization for the effective PEA content of each formulation, PEA-PL supplementation resulted in up to a five-fold greater increase in plasma PEA concentrations relative to baseline after 2 weeks.

### 3.3. Clinical Efficacy in Chronic Neuropathic Low Back Pain

A total of 120 participants were enrolled and randomized equally into three groups (Figure S1): placebo (*n* = 40), PEA-PL 600 → 300 mg (*n* = 40), and PEA-PL 450 mg (*n* = 40). All randomized participants completed the 8-week study period, and no withdrawals or losses to follow-up were recorded. Baseline demographic characteristics and vital parameters were comparable across the three groups (Table 3). The mean age was 45.2 ± 1.3 years in the placebo group, 44.6 ± 1.2 years in the PEA-PL 600 → 300 mg group, and 45.7 ± 1.2 years in the PEA-PL 450 mg group (Kruskal–Wallis test, *p* = 0.578). Gender distribution was balanced, with females comprising approximately 50% of participants in each arm (χ^2^ test, *p* = 0.967). No significant between-group differences were observed in BMI; Kruskal–Wallis test, *p* = 0.715. Likewise, systolic and diastolic blood pressure, heart rate, respiratory rate, and body temperature did not differ significantly between groups at baseline (Kruskal–Wallis tests, all *p* ≥ 0.658), and categorical analyses of these parameters confirmed the absence of baseline imbalances across treatment arms (χ^2^ or Fisher’s exact tests, all *p* ≥ 0.62).

#### 3.3.1. PEA-PL Supplementation Effect on Primary Outcome Measure

##### Effect on Chronic LBP (Neuropathic Pain) (DN4 Score)

Mixed-effects analysis revealed significant effects of treatment group, visit, and their interaction on DN4 scores (all *p* < 0.0001) after adjustment for gender, age, and BMI. Gender showed a modest association with DN4 scores (*p* = 0.0498), whereas age and BMI categories were not significant covariates (Table S1).

**Figure 2:**
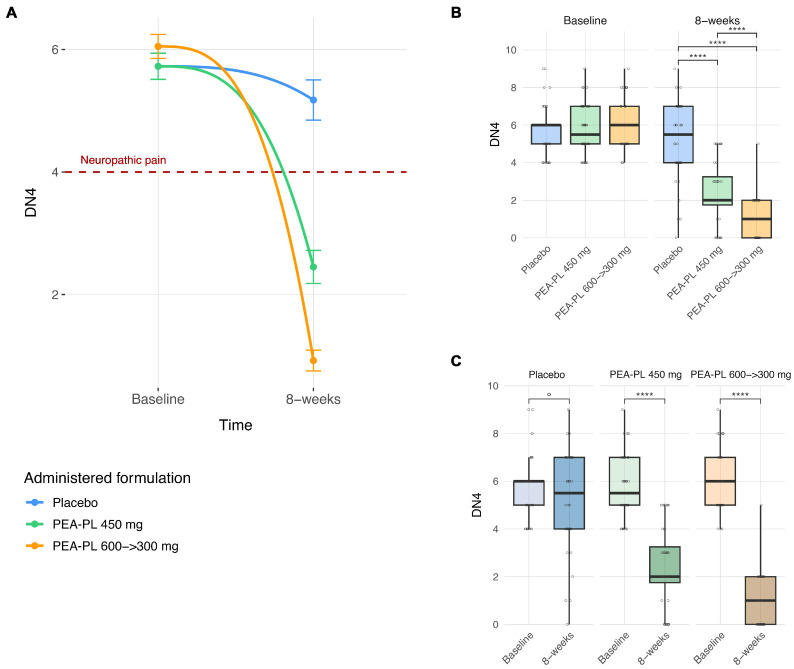
Effect of PEA-PL supplementation on DN4 neuropathic pain scores over 8 weeks. (**A**) Connected scatterplots show mean DN4 scores (±SEM) at baseline and 8 weeks in the placebo, PEA-PL 600 → 300 mg, and PEA-PL 450 mg groups. Smooth trend lines were fitted using cubic B-spline regression, providing a flexible interpolation of the observed means. The horizontal reference line indicates the diagnostic threshold for neuropathic pain (DN4 ≥ 4). (**B**) Boxplots illustrate between-group comparisons at baseline and at 8 weeks. (**C**) Boxplots display within-group changes from baseline to 8 weeks. *p*-values were derived from linear mixed-effects models adjusted for gender, age, and BMI, with false discovery rate (FDR) correction. Significance levels: ° *p* ≤ 0.1; **** *p* ≤ 0.0001.

At the 8-week follow-up, between-group comparisons demonstrated significantly lower DN4 scores in both PEA-PL groups compared with placebo (Figure 2A,B). DN4 scores were reduced by 2.80 points in the PEA-PL 450 mg group versus placebo (*p* < 0.0001) and by 4.26 points in the PEA-PL 600 → 300 mg group versus placebo (*p* < 0.0001). In addition, the step-up dosing regimen produced significantly greater improvement than the 450 mg formulation (difference −1.46 points; *p* < 0.0001).

Within-group analyses showed a progressive decline in DN4 scores over the 8-week supplementation period, with substantial differences in magnitude between treatment arms (Figure 2A,C). Participants receiving placebo exhibited only a small, non-significant reduction in DN4 score (−0.55 points; *p* = 0.071). In contrast, both PEA-PL regimens were associated with marked and statistically significant reductions: −3.28 points with PEA-PL 450 mg (*p* < 0.0001) and −5.13 points with the PEA-PL 600 → 300 mg regimen (*p* < 0.0001).

Clinically, these changes resulted in pain relief in most participants in the PEA-PL groups falling below the diagnostic threshold for neuropathic pain (DN4 ≥ 4) at study end, whereas the majority of placebo-treated participants remained above this cutoff.

##### Effect on Pain Severity (NPRS Score)

Mixed-effects analysis revealed significant effects of treatment group, visit, and their interaction on NPRS scores (all *p* < 0.0001), after adjustment for gender, age, and BMI. None of these demographic covariates showed a significant association with NPRS scores (Supplementary Table S1).

**Figure 3:**
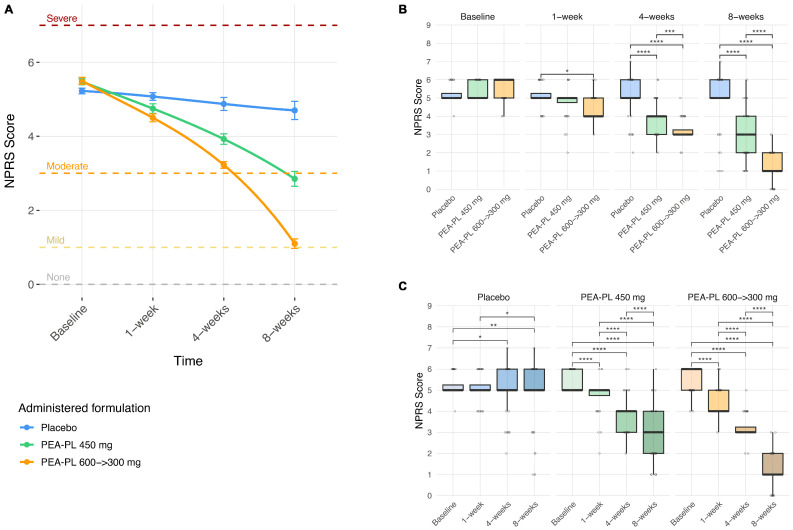
Effect of PEA-PL supplementation on pain intensity (NPRS) over 8 weeks. (**A**) Connected scatterplots show mean NPRS scores (±SEM) at baseline, 1 week, 4 weeks, and 8 weeks in the placebo, PEA-PL 600 300 mg, and PEA-PL 450 mg groups. Smooth trend lines were fitted using cubic B-spline regression, providing a flexible interpolation of the observed means. Horizontal dashed lines indicate clinical pain categories: no pain (0), mild pain (1–3), moderate pain (4–6), and severe pain (7–10). (**B**) Boxplots illustrate between-group comparisons at each timepoint. (**C**) Boxplots display within-group changes from baseline across follow-up visits. *p*-values were derived from linear mixed-effects models adjusted for gender, age, and BMI, with false discovery rate (FDR) correction. Significance levels: * *p* ≤ 0.05; ** *p* ≤ 0.01; *** *p* ≤ 0.001; **** *p* ≤ 0.0001.

At the between-group level, differences emerged early during follow-up (Figure 3A,B). At Week 1, NPRS scores were significantly lower in the PEA-PL 600 → 300 mg group compared with placebo (−0.59 points; *p* = 0.013), whereas the PEA-PL 450 mg group did not differ significantly from placebo (*p* = 0.14). By Week 4, both PEA-PL regimens were superior to placebo, with mean reductions of −0.97 points for PEA-PL 450 mg and −1.66 points for PEA-PL 600 → 300 mg (both *p* < 0.0001); the step-up regimen also produced greater reductions than the 450 mg dosage (−0.70 points; *p* = 0.0008). At Week 8, between-group differences further increased: NPRS scores were lower by −1.87 points in the PEA-PL 450 mg group and −3.61 points in the PEA-PL 600 → 300 mg group compared with placebo (both *p* < 0.0001), with the step-up regimen again outperforming the 450 mg dose (−1.75 points; *p* < 0.0001).

Within-group analyses revealed progressive reductions in pain intensity over the 8-week supplementation period (Figure 3A,C). In the placebo arm, reductions were modest and reached statistical significance only at Week 4 (−0.35 points; *p* = 0.034) and Week 8 (−0.53 points; *p* = 0.002) relative to baseline. In contrast, both PEA-PL regimens were associated with earlier and larger reductions in NPRS scores. The PEA-PL 450 mg group showed significant improvement as early as Week 1 (−0.73 points; *p* < 0.0001), which increased at Week 4 and Week 8 (−1.55 and −2.63 points, respectively; both *p* < 0.0001). The PEA-PL 600 → 300 mg regimen produced the greatest reductions, with decreases observed at Week 1 (−1.00 points; *p* < 0.0001), Week 4 (−2.28 points; *p* < 0.0001), and Week 8 (−4.40 points; *p* < 0.0001).

Overall, NPRS scores decreased earlier and to a greater extent in participants receiving PEA-PL compared with placebo, with the largest reductions observed in the PEA-PL 600 → 300 mg group over the 8-week study period.

#### 3.3.2. PEA-PL Supplementation Effect on Secondary Outcome Measure

##### Effect on Functional Disability (ODI Score)

Mixed-effects analysis revealed significant effects of treatment group, visit, and their interaction on ODI scores (all *p* < 0.0001), after adjustment for gender, age, and BMI. Gender showed a modest association with ODI scores (*p* = 0.052), whereas age and BMI categories were not significant covariates (Supplementary Table S1).

**Figure 4:**
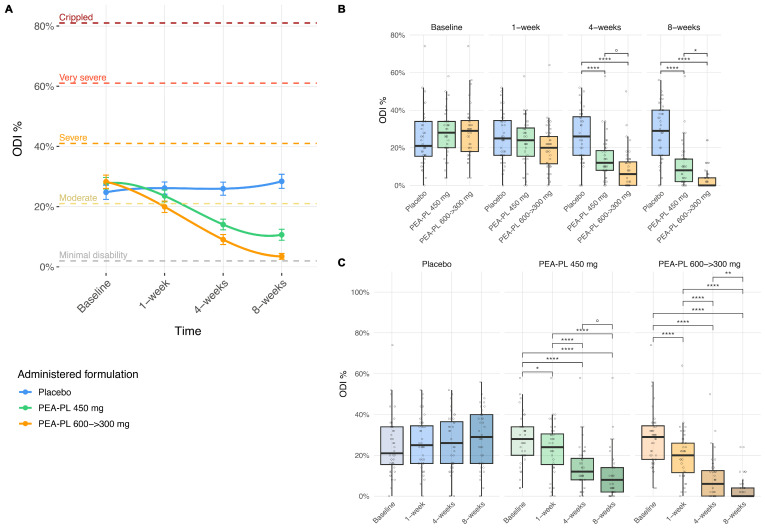
Effect of PEA-PL supplementation on Oswestry Disability Index (ODI) scores over 8 weeks. (**A**) Connected scatterplots show mean ODI scores (±SEM) at baseline, 1 week, 4 weeks, and 8 weeks in the placebo, PEA-PL 600 → 300 mg, and PEA 450 mg groups. Smooth trend lines were fitted using cubic B-spline regression, providing a flexible interpolation of the observed means. Horizontal dashed lines indicate established disability categories: minimal disability (≤20%), moderate disability (21–40%), severe disability (41–60%), very severe disability (61–80%), and crippled (≥81%). (**B**) Boxplots illustrate between-groups comparisons at each timepoint. (**C**) Boxplots display within-group changes from baseline across follow-up visits. *p*-values were derived from linear mixed-effects models adjusted for gender, age, and BMI, with false discovery rate (FDR) correction. Significance levels: ° *p* ≤ 0.1; * *p* ≤ 0.05; ** *p* ≤ 0.01; **** *p* ≤ 0.0001.

At the between-group level (Figure 4A,B), no differences were observed at baseline (*p* ≥ 0.43). At Week 1, a trend toward improvement was observed in the PEA-PL 600 → 300 mg group compared with placebo (−0.06; *p* = 0.01), whereas no significant differences were detected for the PEA-PL 450 mg group. By Week 4, both PEA-PL regimens were superior to placebo, with mean differences of −0.12 and −0.17 for the 450 mg and 600 → 300 mg groups, respectively (both *p* < 0.0001); the step-up regimen also tended to produce greater improvement than the 450 mg dose (−0.05; *p* = 0.069). At Week 8, between-group contrasts further increased: reductions were −0.18 for PEA-PL 450 mg versus placebo (*p* < 0.0001) and −0.25 for PEA-PL 600 → 300 mg versus placebo (*p* < 0.0001), with the step-up regimen again showing significantly greater improvement than the 450 mg dose (−0.07; *p* = 0.010).

Within-group analyses showed progressive reductions in functional disability over the 8-week supplementation period (Figure 4A,C). The placebo group showed no significant changes at any time point (*p* ≥ 0.23). In contrast, the PEA-PL 450 mg group demonstrated gradual improvement, with reductions of −0.04 at Week 1 (*p* = 0.022), −0.14 at Week 4, and −0.17 at Week 8 (both *p* < 0.0001). The PEA-PL 600 → 300 mg step-up regimen produced the largest reductions, with significant improvement already at Week 1 (−0.08), which increased at Week 4 (−0.19) and reached −0.25 at Week 8 (all *p* < 0.0001).

At study end, ODI scores in the PEA-PL groups were lower than at baseline and lower than in the placebo group, whereas scores in the placebo arm remained largely unchanged over time.

##### Effect on Sleep Disturbance (PSQI Score)

Mixed-effects analysis demonstrated significant effects of treatment group, visit, and their interaction on PSQI scores (all *p* < 0.0001), after adjustment for gender, age, and BMI. Among covariates, BMI category showed a modest but statistically significant association with PSQI scores (*p* = 0.029), whereas gender and age were not significant (Supplementary Table S1).

**Figure 5:**
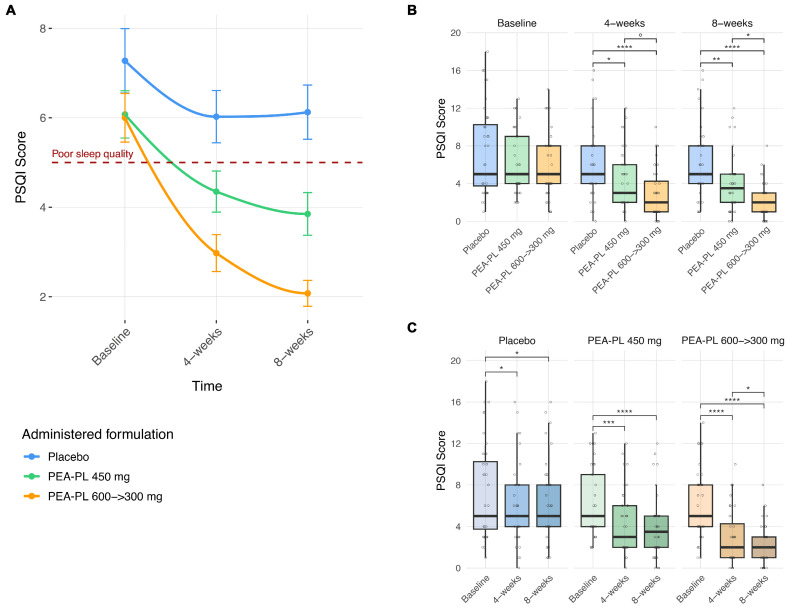
Effect of PEA-PL supplementation on the Pittsburgh Sleep Quality Index (PSQI )scores over 8 weeks. (**A**) Connected scatterplots show mean PSQI scores (±SEM) at baseline, 4 weeks, and 8 weeks in the placebo, PEA-PL 600 → 300 mg, and PEA-PL 450 mg groups. Smooth trend lines were added using shape-preserving cubic interpolation (PCHIP), ensuring the curve passes through observed means. The horizontal dashed line indicates the clinical cutoff for poor sleep quality (PSQI ≥ 5). (**B**) Boxplots illustrate between-group comparisons at each timepoint. (**C**) Boxplots display within-group changes from baseline to follow-up. *p*-values were derived from linear mixed-effects models adjusted for gender, age, and BMI, with false discovery rate (FDR) correction. Significance levels: ° *p* ≤ 0.1; * *p* ≤ 0.05; ** *p* ≤ 0.01; *** *p* ≤ 0.001; **** *p* ≤ 0.0001.

At the between-group level (Figure 5A,B), no differences were observed at baseline (*p* ≥ 0.11). At Week 4, both PEA-PL regimens were associated with significantly lower PSQI scores compared with placebo, with mean differences of −1.82 points for PEA-PL 450 mg (*p* = 0.024) and −3.19 points for PEA-PL 600 → 300 mg (*p* < 0.0001); the step-up regimen also tended to produce greater improvement than the 450 mg dose (−1.38 points; *p* = 0.069). At Week 8, between-group differences increased further: PSQI scores were lower by −2.42 points in the PEA-PL 450 mg group (*p* = 0.002) and by −4.19 points in the PEA-PL 600 → 300 mg group (*p* < 0.0001) compared with placebo, with the step-up regimen showing significantly greater improvement than the 450 mg dose (−1.78 points; *p* = 0.019).

Within-group analyses revealed progressive reductions in PSQI scores over the 8-week supplementation period (Figure 5A,C). In the placebo group, PSQI scores decreased modestly, with significant reductions observed at Week 4 (−1.25 points; *p* = 0.018) and Week 8 (−1.15 points; *p* = 0.018) relative to baseline. In contrast, both PEA-PL regimens were associated with larger and more consistent reductions. The PEA-PL 450 mg group showed decreases of −1.73 points at Week 4 (*p* = 0.0003) and −2.23 points at Week 8 (*p* < 0.0001), whereas the PEA-PL 600 → 300 mg regimen produced the greatest reductions, with −3.03 points at Week 4 and −3.93 points at Week 8 (both *p* < 0.0001).

At study end, PSQI scores in both PEA-PL groups were lower than at baseline and lower than in the placebo group, with a greater proportion of participants in the PEA-PL arms falling below the PSQI threshold of ≥5 for poor sleep quality.

##### Effect on Health-Related Quality of Life (QoL PCS, MCS Scores)

Mixed-effects analysis demonstrated significant effects of treatment group, visit, and their interaction on QoL both the PCS and MCS scores (all *p* < 0.0001), after adjustment for gender, age, and BMI (Supplementary Table S1). None of these demographic covariates showed a significant association with QoL outcomes.

**Figure 6:**
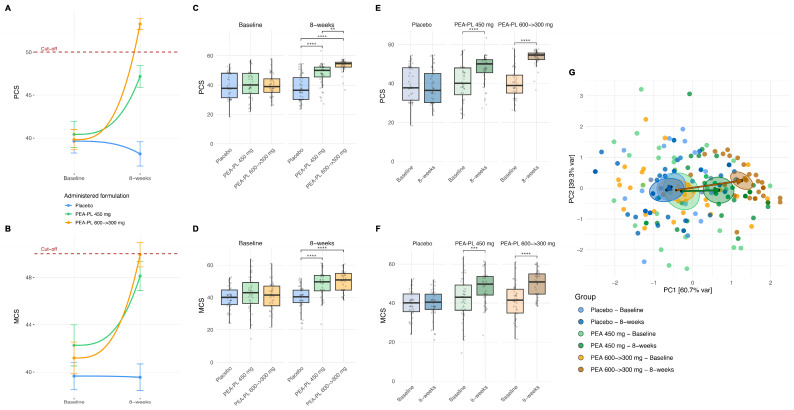
Effect of PEA-PL supplementation on the Quality of Life (QoL) PCS and PCS scores over 8 weeks. Connected scatterplots show mean PCS (**A**) and MCS (**B**) scores (±SEM) at baseline and 8 weeks in the placebo, PEA-PL 600 → 300 mg, and PEA-PL 450 mg groups. The horizontal dashed line indicates the U.S. normative range (50 ± 10). (**C**,**D**) Boxplots illustrate between-group comparisons at baseline and 8 weeks for PCS and MCS, respectively. (**E**,**F**) Boxplots display within-group changes from baseline to 8 weeks for PCS and MCS. (**G**) Principal component analysis (PCA) based of PCS and MCSshowing baseline and 8-week centroids for each treatment group (placebo, PEA-PL 450 mg, and PEA-PL 600 → 300 mg) connected by arrows, projected onto the first two principal components (PC1 and PC2), which capture the largest proportion of variance in combined physical and mental health measures. In this plot,rightward shifts indicate global improvement in both PCS and MCS, while vertical displacement reflects whether the relative contribution to QoL improvement was greater for mental health (upward) or for physical health (downward). *p*-values were derived from linear mixed-effects models adjusted for gender, age, and BMI, with false discovery rate (FDR) correction. PCA group separations were tested using PERMANOVA (adonis2, 9999 permutations). Significance levels: ** *p* ≤ 0.01; *** *p* ≤ 0.001; **** *p* ≤ 0.0001.

At the between-group level (Figure 6A–D), no differences were observed at baseline for either PCS or MCS scores (*p* ≥ 0.33). At Week 8, both PEA-PL regimens were associated with significantly greater improvements in physical and mental health scores compared with placebo. For PCS (Figure 6C), scores were higher by 9.18 points in the PEA-PL 450 mg group and by 14.60 points in the PEA-PL 600 → 300 mg group relative to placebo (both *p* < 0.0001), with the step-up regimen also producing greater improvement than the 450 mg dose (difference + 5.42 points; *p* = 0.0042). For MCS (Figure 6D), similar patterns were observed, with increases of 9.10 points for the PEA-PL 450 mg group and 10.68 points for the PEA-PL 600 → 300 mg group compared with placebo (both *p* < 0.0001), while no significant difference was detected between the two PEA-PL regimens.

Within-group analyses confirmed these findings (Figure 6E,F). PCS scores remained stable in the placebo group (*p* = 0.37), whereas significant increases were observed in both PEA-PL groups: +6.91 points in the 450 mg group and +13.33 points in the 600 → 300 mg group (both *p* < 0.0001). Similarly, MCS scores did not change significantly in the placebo arm (*p* = 0.65), while both PEA-PL regimens showed significant improvements, with increases of +6.02 points in the 450 mg group (*p* = 0.0002) and +8.39 points in the 600 → 300 mg group (*p* < 0.0001).

Principal component analysis (PCA) of PCS and MCS scores supported these results (Figure 6G). Within-group trajectories showed significant shifts from baseline to Week 8 in both PEA-PL arms (PERMANOVA, *p* = 0.0002), whereas no significant change was observed in the placebo group (*p* = 0.81). At Week 8, both PEA-PL regimens clustered distinctly from placebo (*p* = 0.0003), with partial separation between the step-up regimen and the 450 mg dose (*p* = 0.0009).

At study end, mean PCS and MCS scores were higher in both PEA-PL groups than at baseline and higher than in the placebo group, whereas scores in the placebo arm remained largely unchanged.

##### Effect on Concomitant Pain Medication Use

**Figure 7:**
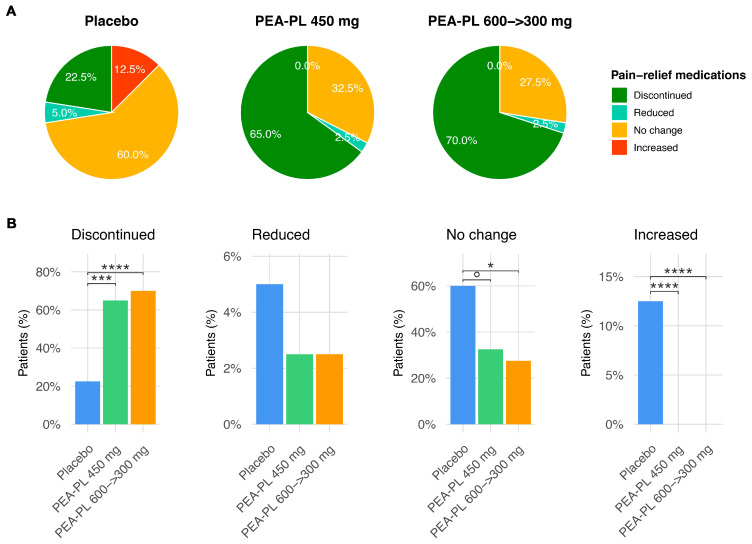
Impact of PEA-PL supplementation on concomitant analgesic use. (**A**) Pie charts show the percentage distribution of changes in concomitant pain medication from baseline to 8 weeks within each treatment arm (Placebo, PEA-PL 450 mg, PEA-PL 600 → 300 mg). Categories: discontinued medication, reduced medication, no change, and increased medication. (**B**) Bar plots comparing the proportion of subjects in each category across treatment arms. Statistical annotations reflect pairwise contrasts from the multinomial logistic regression model (FDR-corrected *p*-values). Significance codes: ° *p* ≤ 0.1; * *p* ≤ 0.05; *** *p* ≤ 0.001; **** *p* ≤ 0.0001.

Changes in concomitant analgesic use differed significantly among treatment arms (Figure 7). Discontinuation of analgesic medication occurred in 22.5% of participants in the placebo group, compared with 65.0% and 70.0% in the PEA-PL 450 mg and PEA-PL 600 → 300 mg groups, respectively (*p* ≤ 0.0001). The proportion of participants with unchanged analgesic regimens was highest in the placebo group (60.0%), compared with 32.5% in the PEA-PL 450 mg group and 27.5% in the PEA-PL 600 → 300 mg group; the comparison between placebo and the 600 → 300 mg regimen reached statistical significance (*p* = 0.028), whereas the comparison with the 450 mg group showed a non-significant trend (*p* = 0.073).

The proportion of participants classified in the “reduced medication” category was similar across groups (2.5–5.0%), with no statistically significant differences. In contrast, increases in analgesic use were observed exclusively in the placebo group (12.5%), which was significantly higher than in both PEA-PL treatment arms (*p* < 0.0001).

#### 3.3.3. Safety Assessment

##### Adverse Effects

No serious adverse events or treatment discontinuations were reported during the trial. Safety was primarily assessed through adverse event monitoring (Table S2). Both PEA-PL dose regimens were well tolerated, with only mild and transient adverse events observed.

In the PEA-PL 600 → 300 mg group, six participants reported adverse effects, including mild constipation, transient fatigue, leg discomfort, sleep disturbance, or short-lived morning back pain. In the PEA-PL 450 mg group, six participants reported isolated cases of drowsiness, mild heartburn, irritability, or leg discomfort. In the placebo group, seven participants reported adverse events, most commonly leg discomfort, as well as mild gastrointestinal symptoms such as nausea and stomach upset.

##### Vital Signs

Vital sign analyses further supported the overall safety profile. Mixed-effects modeling revealed no consistent treatment-related differences across study arms. BMI category showed a modest association with systolic and diastolic blood pressure (*p* ≤ 0.035), whereas no significant associations were observed for age or gender (Table S1).

Between-group comparisons (Figure S2A,B) identified a small increase in body temperature at Week 8 in the placebo group (Δ = +0.42 to +0.46 °C; *p* < 0.0001) compared with both PEA-PL groups; however, all recorded values remained within the clinically normal range (35–38 °C). No statistically significant between-group differences were observed for systolic or diastolic blood pressure, heart rate, or respiratory rate.

Within-group analyses (Figure S2A,C) showed that vital signs remained generally stable over the 8-week study period. A modest reduction in heart rate was observed across all groups (Δ −3.73 to −2.05 bpm; *p* ≤ 0.0035). Respiratory rate exhibited a small decrease in the PEA-PL groups (Δ −0.8 breaths/min; *p* ≤ 0.042), without evidence of group-specific effects. Minor changes in body temperature were observed only in the placebo group (Δ +0.57 °C; *p* < 0.0001). Shift analysis confirmed these findings, with most participants remaining stable across time points and only a small proportion exhibiting minor changes, without consistent differences between treatment arms (Table S3).

Overall, no clinically meaningful alterations in vital signs or consistent treatment-related safety signals were observed during the 8-week PEA-PL supplementation period.

## 4. Discussion

In this study, a food-grade, phospholipid-delivery system-based formulation of PEA was investigated for its apparent solubility, systemic exposure, and clinical effects in chronic neuropathic LBP. The phospholipid-based system increased PEA solubility approximately eight-fold in FaSSIF compared with unformulated PEA, addressing a key limitation related to its poor aqueous dispersibility. Consistent with this improvement, plasma concentration study in healthy volunteers demonstrated that PEA-PL achieved higher and more sustained plasma PEA concentrations than unformulated PEA, despite a lower administered dose, supporting the role of the phospholipid delivery system in facilitating stability, intestinal dispersion and systemic availability.

These bioabsorption advantages translated into clinically meaningful benefits in the double-blind, placebo-controlled randomized clinical trial conducted in adults with chronic neuropathic LBP. After 8 weeks of supplementation, PEA-PL was associated with greater improvements than placebo across multiple validated clinical domains encompassing pain severity, functional disability, sleep disturbance, and health-related QoL, with the most pronounced effects observed for the step-down 600 → 300 mg regimen. When expressed as baseline-adjusted relative differences versus placebo (ratio-of-change, ROC; Table S4), the 600 → 300 mg regimen yielded marked reductions in neuropathic pain (DN4, −82.5%; NPRS, −78.2%), functional disability (ODI, −90.4%), and sleep disturbance (PSQI, −55.6%), together with substantial improvements in health-related both physical and mental components of QoL (PCS, +37.8%; MCS, +21.1%). Overall, the convergence of an eight-fold increase in solubility, optimized systemic exposure, and substantial multi-domain clinical benefits supports the biological plausibility of the observed outcomes and is consistent with demonstrated PEA’s multimodal mechanisms of action—including modulation of neuroinflammation and nociceptive signaling—as well as with existing clinical evidence [15,16,20].

Across both clinical studies, PEA-PL supplementation was well tolerated, with no serious adverse events and no clinically meaningful alterations in vital signs. The frequency and nature of mild adverse events were comparable between the PEA-PL and placebo groups, in line with the extensive preclinical and clinical literature indicating that PEA lacks major safety concerns [15,19,22].

Taken together, these findings support phospholipid-based PEA supplementation as a supportive, analgesic, and anti-inflammatory add-on therapy for chronic neuropathic LBP, integrated within multimodal management strategies to improve pain control, functional capacity, and quality of life while maintaining favorable tolerability.

The PEA-PL efficacy outcomes observed in the present study on chronic LPB are consistent with, and extend, the existing clinical evidence supporting the use of PEA in neuropathic pain. PEA has been employed in the management of neuropathic pain for more than two decades [46], owing to its demonstrated anti-inflammatory and analgesic properties. Several randomized controlled trials have investigated oral PEA supplementation in chronic neuropathic LBP as well as in other neuropathic pain conditions, including lumbosciatica, carpal tunnel syndrome, diabetic polyneuropathy, postherpetic neuralgia, and chemotherapy-induced neuropathy [18,19,20,22]. Collectively, these studies have reported reductions in pain intensity, improvements in functional outcomes, and favorable tolerability. Consistent with these findings, a systematic review and meta-analysis of double-blind randomized controlled trials confirmed PEA’s efficacy and safety in chronic pain management while highlighting substantial heterogeneity in dosing regimens, ranging from 300 to 1200 mg/day [20]. Notably, in a large clinical study including 636 subjects with sciatic pain, PEA administered at doses of 300 or 600 mg/day for three weeks resulted in significant improvements in pain intensity and functional disability [47]. The estimated number needed to treat (NNT) for achieving a ≥50% pain reduction was approximately 1.5, indicating a magnitude of effect comparable to, or greater than, that reported for several standard co-analgesic therapies. In this context, the present study extends prior evidence by demonstrating clinically meaningful, multi-domain benefits of a novel phospholipid-based PEA formulation in subjects with chronic neuropathic LBP.

Several formulation strategies have been explored to address the intrinsic limitations of PEA, particularly its lipophilicity and limited dissolution in gastrointestinal fluids. Prior approaches have primarily focused on particle-size reduction (micronized and ultramicronized PEA) to increase dissolution rate and reduce variability of absorption, with clinical use reported across multiple neuropathic and chronic pain settings [23]. More recently, alternative delivery concepts such as lipid-based encapsulation (e.g., liposomal or nanocarrier systems) and self-emulsifying/hydrogel-type formulations have also been described, largely aiming to enhance oral bioaccessibility and systemic exposure [48].

In this context, the present work evaluates a phospholipid-based formulation of PEA developed using Phytosome™ technology (Cronilief™) and provides preliminary human evidence linking formulation to systemic exposure and to clinical outcomes in chronic neuropathic LBP. While head-to-head comparisons across different PEA formulations are limited and beyond the scope of the present study, the integrated bioabsorption–clinical design supports the translational premise that phospholipid formulation can optimize systemic exposure and may contribute to clinically meaningful benefits, thereby addressing the design of future comparative and mechanistic investigations.

Endogenously, PEA is synthesized on demand from membrane phospholipids in response to cellular stress or injury and accumulates in both peripheral tissues and central nervous system regions involved in nociception, including the spinal cord and brain [16]. Chronic inflammatory states have been associated with reduced endogenous PEA levels [17,22], suggesting that exogenous supplementation may help restore physiological concentrations and re-establish its protective functions. Mechanistically, many of PEA’s effects are mediated through activation of the PPAR-α, which regulates transcriptional pathways involved in inflammation and pain modulation [49]. Additional mechanisms include modulation of transient receptor potential vanilloid 1 (TRPV1) channels, indirect enhancement of cannabinoid CB1 and CB2 receptor signaling via inhibition of anandamide degradation (the “entourage effect”), and engagement of G protein–coupled receptors such as GPR55 and GPR119 [18]. Beyond receptor-level actions, PEA modulates immune responses by inhibiting mast cell activation and microglial reactivity, processes that are particularly relevant to the pathophysiology of neuropathic pain [18,19,20,22].

The present research has several limitations that should be acknowledged. Both clinical studies were designed as exploratory investigations. The bioabsorption assessment in healthy volunteers was intended as a proof-of-concept evaluation of the phospholipid-based delivery system, focusing on solubility optimization and systemic exposure rather than definitive pharmacokinetic characterization. Similarly, the randomized clinical trial in adults with chronic neuropathic LBP was conducted to explore clinical efficacy signals and tolerability within a controlled setting and predefined intervention period, precluding the assessment of long-term efficacy and safety. Neuroinflammatory and pain-related biomarkers were not included, as the primary objective of the study was to establish preliminary clinical relevance and feasibility rather than mechanistic confirmation. Furthermore, the single-center design of the clinical efficacy trial may limit the generalizability of the findings. Collectively, these limitations may address the design of future studies, which should include larger, adequately powered, multicenter randomized controlled trials with extended follow-up and integrated biomarker analyses to more fully define the supportive clinical and mechanistic role of phospholipid-based PEA formulation in chronic neuropathic pain.

## 5. Conclusions

The phospholipid-based delivery system (Phytosome™) represented an effective approach to optimize the solubility and systemic exposure of supplemental PEA (Cronilief™), which was associated with clinically meaningful improvements in pain severity, functional disability, and overall subject-reported outcomes in chronic neuropathic LBP.

## Figures and Tables

**Table 1:** Solubility study of Phospholipid-based PEA (PEA-PL) and unformulated PEA in FaSSIF, pH 6.5.

Material	PEA (mg/mL)
PEA	0.016 ± 0.003
PEA-PL	0.133 ± 0.020

Data are reported as mean ± SD. FaSSIF: fasted-state simulated intestinal fluid.

**Table 2:** Plasma palmitoylethanolamide (PEA) concentrations determined by LC–MS/MS during 2-week supplementation.

Supplement	Total Daily PEA Dose (mg)	PEA Content (mg)	PEA Plasma Conc. (pg/mL)			Variation (%)	
			T0	T1	T2	T1 vs. T2	T2 vs. T
**PEA**	300	300 (100%)	1797.8 ± 127.7	2459.9 * ± 409.8	3024.0 * ± 900.1	+37	+68
**PEA-PL**	300	120 (40%)	1727.1 ± 144.2	2168.4 * ± 174.0	3366.5 ** ± 901.6	+26	+95
**PEA-PL**	600	240 (40%)	1963.2 ± 100.0	3244.5 ** ± 415.7	3264.6 * ± 551.2	+65	+66

PEA concentrations are reported as mean ± SEM at baseline (T0), 2 h after the first dose (T1), and 2 h after the last dose of the 14-day supplementation period (T2). In the PEA-PL, the declared PEA content corresponds to 40% (*w*/*w*) of the total formulation weight. * *p* < 0.05 and ** *p* < 0.01, by an intra-group Wilcoxon test vs. baseline level.

**Table 3:** Participants’ demographic and baseline vital characteristics by treatment group.

Section	Variable	Placebo	PEA-PL 450 mg	PEA-PL 600 -> 300 mg	*p*-Value
**Demographics**	**Participants, *n***	*n* = 40	*n* = 40	*n* = 40	
	**Age (years)**	45.23 ± 1.28	45.73 ± 1.22	44.58 ± 1.17	0.578
	*Category*				0.776
	40 < Age ≤ 50	18 (45.0%)	20 (50.0%)	18 (45.0%)	
	50 < Age ≤ 60	7 (17.5%)	9 (22.5%)	6 (15.0%)	
	Age ≤ 40	15 (37.5%)	11 (27.5%)	16 (40.0%)	
	**Gender**				0.967
	F	20 (50.0%)	20 (50.0%)	19 (47.5%)	
	M	20 (50.0%)	20 (50.0%)	21 (52.5%)	
	**BMI (kg/m^2^)**	31.10 ± 0.71	30.02 ± 0.96	30.76 ± 0.95	0.715
	*Category*				0.258
	Normal weight	3 (7.5%)	8 (20.0%)	7 (17.5%)	
	Obesity C1	10 (25.0%)	6 (15.0%)	13 (32.5%)	
	Obesity C2	11 (27.5%)	13 (32.5%)	10 (25.0%)	
	Obesity C3	1 (2.5%)		2 (5.0%)	
	Overweight	15 (37.5%)	12 (30.0%)	7 (17.5%)	
	Underweight		1 (2.5%)	1 (2.5%)	
**Vitals**	**Systolic BP (mmHg)**	116.95 ± 2.39	115.00 ± 1.79	116.25 ± 2.11	0.845
	*Category*				0.618
	High (≥120)	13 (32.5%)	12 (30.0%)	16 (40.0%)	
	Normal	27 (67.5%)	28 (70.0%)	24 (60.0%)	
	**Diastolic BP (mmHg)**	68.65 ± 2.19	69.50 ± 1.86	67.95 ± 1.56	0.720
	*Category*				0.734
	High (≥80)	8 (20.0%)	11 (27.5%)	10 (25.0%)	
	Low (<60)	1 (2.5%)	1 (2.5%)	3 (7.5%)	
	Normal	31 (77.5%)	28 (70.0%)	27 (67.5%)	
	**Heart rate (bpm)**	81.62 ± 1.42	81.28 ± 1.50	81.55 ± 1.61	0.994
	*Category*				1.000
	High (>100)	1 (2.5%)			
	Normal	39 (97.5%)	40 (100.0%)	40 (100.0%)	
	**Respiratory rate (/min)**	15.05 ± 0.47	15.55 ± 0.49	15.40 ± 0.44	0.721
	*Category*				1.000
	High (>20)	3 (7.5%)	3 (7.5%)	3 (7.5%)	
	Low (<12)	1 (2.5%)			
	Normal	36 (90.0%)	37 (92.5%)	37 (92.5%)	
	**Body temperature (°C)**	36.16 ± 0.03	36.22 ± 0.04	36.2 ± 0.04	0.658
	*Category*				NA
	Normal	40 (100.0%)	40 (100.0%)	40 (100.0%)	

Values are presented as mean ± SEM for continuous variables and as counts (percentages) for categorical variables. Between-group comparisons were performed using the Kruskal–Wallis test for continuous variables and χ^2^ or Fisher’s exact tests for categorical variables, as appropriate. No statistically significant differences were observed across treatment arms for baseline demographic characteristics or vital parameters. Body mass index (BMI) categories were defined according to World Health Organization criteria: underweight (<18.5 kg/m^2^), normal weight (18.5–24.9 kg/m^2^), overweight (25.0–29.9 kg/m^2^), obesity class I (30.0–34.9 kg/m^2^), class II (35.0–39.9 kg/m^2^), and class III (≥40.0 kg/m^2^). Abbreviations: BMI, body mass index; BP, blood pressure; NA, not available.

## Data Availability

The original contributions presented in this study are included in the article. Further inquiries can be directed to the corresponding authors.

## Supplementary Materials

The following supporting information can be downloaded at: https://www.mdpi.com/article/10.3390/biomedicines14020380/s1, Figure S1: CONSORT flow chart; Figure S2: Effect of PEA Phospholipids supplementation on vital signs over 8 weeks; Table S1: Type III ANOVA results from linear mixed-effects models for questionnaire and vital sign parameters; Table S2: Treatment-emergent adverse events (TEAEs) reported during the 8-week study period; Table S3: Distribution of participants by stability, improvement, or worsening of vital signs from baseline to 8 weeks; Table S4: Estimated marginal means and percent changes for questionnaire scores across visits and treatment arms.
