# Supplementary material for: Phospholipid-Based Delivery System Optimizes the Solubility and Systemic Exposure of Palmitoylethanolamide and Supports Clinical Benefits in Chronic Neuropathic Low Back Pain
Source: Biomedicines. 2026 Feb 6;14(2):380. doi: 10.3390/biomedicines14020380 (PMC12937724; doi:10.3390/biomedicines14020380)
Supplement: Supplementary file 1 [file biomedicines-14-00380-s001.zip › Supplementary Information.pdf]

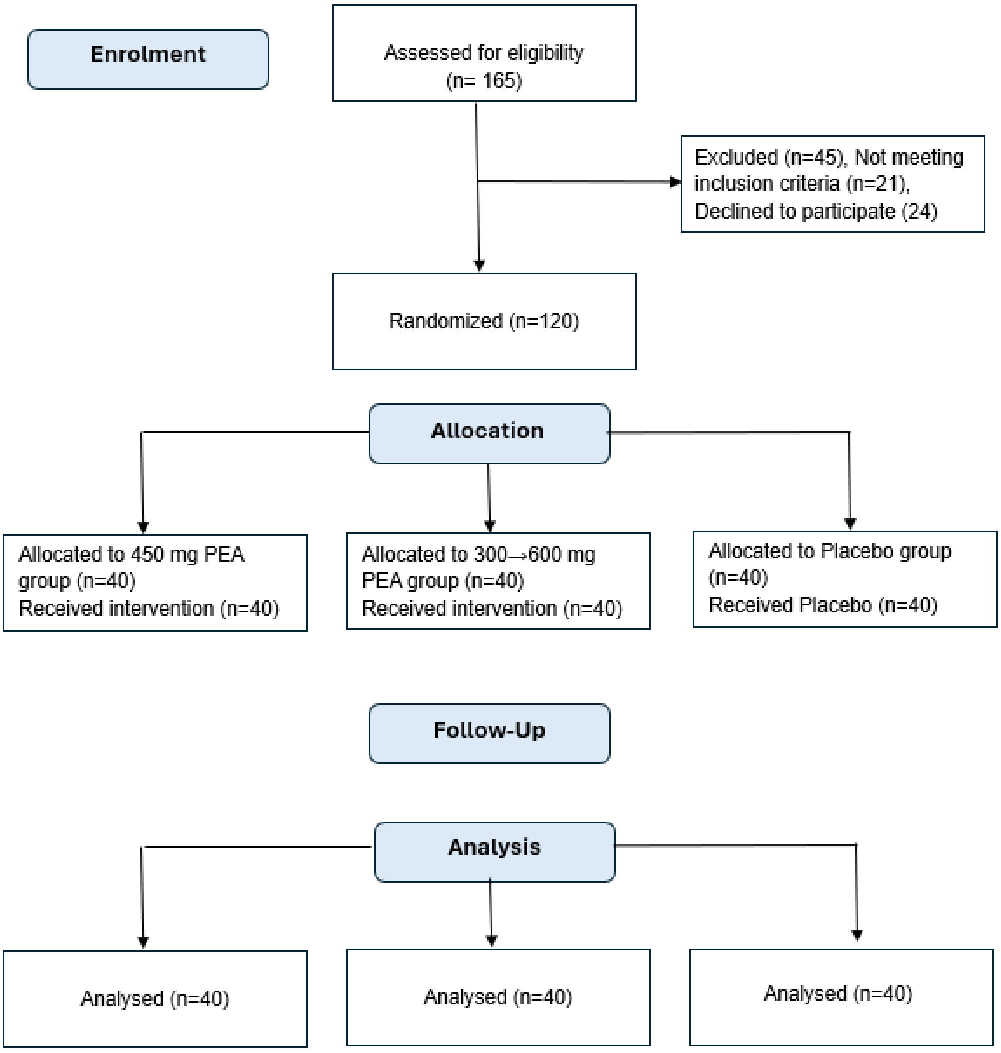

Figure S1. Study CONSORT Flow chart

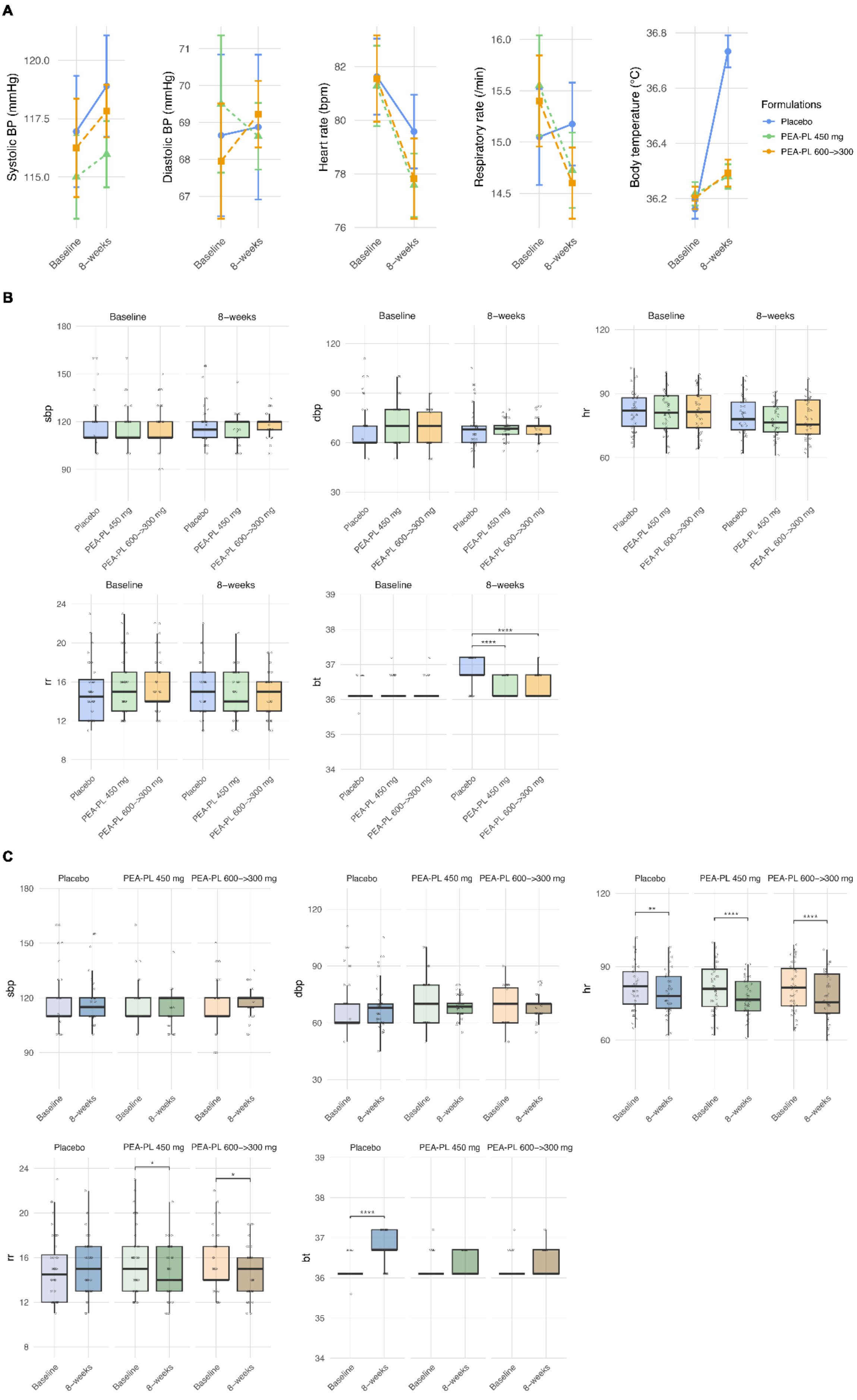
A
Systolic BP (mmHg)
Diastolic BP (mmHg)
Heart rate (bpm)
Respiratory rate (/min)
Body temperature (°C)
Baseline
8–weeks
Formulations
Placebo
PEA-PL 450 mg
PEA-PL 600–>300
B
sbp
dbp
hr
rr
bt
PEA-PL 600–>300 mg
C

Figure S2. Effect of PEA Phospholipids supplementation on vital signs over 8 weeks. (A) Connected scatterplots show mean values (± SEM) for systolic blood pressure (SBP), diastolic blood pressure (DBP), heart rate (HR), respiratory rate (RR), and body temperature (BT) at baseline and 8 weeks in the placebo, PEA-PL 600→300 mg, and PEA-PL 450 mg groups. (B) Boxplots illustrate between-group comparisons at baseline and 8 weeks. (C) Boxplots display within-group changes from baseline to 8 weeks. P-values were derived from linear mixed-effects models adjusted for gender, age, and BMI, with false discovery rate (FDR) correction. Significance levels: ° $p \leq 0.1$; * $p \leq 0.05$; ** $p \leq 0.01$; *** $p \leq 0.001$; **** $p \leq 0.0001$. Abbreviations: SBP, systolic blood pressure; DBP, diastolic blood pressure; HR, heart rate; RR, respiratory rate; BT, body temperature.
